# Protection against stroke with glucagon-like peptide-1 receptor agonists: a comprehensive review of potential mechanisms

**DOI:** 10.1186/s12933-022-01686-3

**Published:** 2022-11-15

**Authors:** Bruno Vergès, Victor Aboyans, Denis Angoulvant, Pierre Boutouyrie, Bertrand Cariou, Fabien Hyafil, Kamel Mohammedi, Pierre Amarenco

**Affiliations:** 1 Department of Endocrinology, Diabetes and Metabolic Disorders, Dijon University Hospital, INSERM Unit, LNC-UMR 1231, University of Burgundy, Dijon, France; 2 Department of Cardiology, EpiMaCT - INSERM UMR, Dupuytren University Hospital, Limoges University, 1094 & IRD 270, Limoges, France; 3 EA4245 Transplantation, Immunity & Inflammation, Department of Cardiology, University of Tours, Tours University Hospital, Tours, France; 4 Paris Cardiovascular Research CenterUMR-970Department of Pharmacology, INSERM, Georges-Pompidou European Hospital, Paris City University, Paris, France; 5 University of Nantes, Nantes University Hospital Centre, CNRS, INSERM, L’institut du Thorax, Nantes, France; 6 Department of Nuclear Medicine, DMU IMAGINA, Georges-Pompidou European Hospital, APHP, Paris City University, Paris, France; 7 Department of Endocrinology, Diabetes, and Nutrition, University of Bordeaux, INSERM U1034, Pessac, France; 8 Neurology and Stroke Center, SOS-TIA Clinic, Bichat Hospital, University of Paris, Paris, France

**Keywords:** Stroke, Glucagon-like peptide-1 receptor agonists, GLP-1, Neuroprotection, Mechanisms

## Abstract

Several randomized controlled trials have demonstrated the benefits of glucagon-like peptide-1 receptor agonists (GLP-1RAs) on ischemic stroke in patients with diabetes. In this review, we summarize and discuss the potential mechanisms of stroke protection by GLP-1RAs. GLP-1RAs exert multiple anti-atherosclerotic effects contributing to stroke prevention such as enhanced plaque stability, reduced vascular smooth muscle proliferation, increased nitric oxide, and improved endothelial function. GLP-1RAs also lower the risk of stroke by reducing traditional stroke risk factors including hyperglycemia, hypertension, and dyslipidemia. Independently of these peripheral actions, GLP-1RAs show direct cerebral effects in animal stroke models, such as reduction of infarct volume, apoptosis, oxidative stress, neuroinflammation, excitotoxicity, blood–brain barrier permeability, and increased neurogenesis, neuroplasticity, angiogenesis, and brain perfusion. Despite these encouraging findings, further research is still needed to understand more thoroughly the mechanisms by which GLP-1RAs may mediate stroke protection specifically in the human diabetic brain.

## Introduction

Glucagon-like peptide-1 (GLP-1) receptor agonists (GLP-1RAs) are becoming a common treatment option for people with type 2 diabetes mellitus (T2DM) due to their various benefits, such as their weight loss benefit and glycemic-lowering efficacy, without increased risk of hypoglycemia. Most notably, a series of large-scale cardiovascular outcome trials (CVOTs) created robust clinical evidence for the cardiovascular benefits of GLP-1RAs in patients with T2DM and high cardiovascular risk or established atherosclerotic cardiovascular disease (ASCVD) [1, 2]. Among those benefits, a protective effect of GLP-1RAs against stroke has been consistently demonstrated [1]. In a 2020 meta-analysis of seven CVOTs, the use of GLP-1RAs was associated with a 15% lower risk of non-fatal stroke, a 19% lower risk of fatal stroke, and a 16% lower risk of total stroke [2]. Consistently, in a more recent, updated meta-analysis of eight CVOTs, GLP-1RAs reduced the risk of fatal or non-fatal stroke by 17% [3]. Likewise, in a large retrospective cohort study of adults with T2DM, treatment with GLP-1RAs (N = 4,460) was associated with a 29% reduction in the risk of non-fatal ischemic stroke when compared to treatment with dipeptidyl peptidase-4 inhibitors (DPP-4i) (N = 13,380) [4]. In this paper, we aimed to summarize and discuss the potential extracerebral and cerebral mechanisms of stroke protection by GLP-1RAs, based on a review of both non-clinical and clinical studies that evaluated the mechanistic effects of GLP-1 and/or GLP-1RAs in preventing ischemic stroke.

## Glucagon-like peptide-1 receptor agonists and glucagon-like peptide-1 receptors

GLP-1RAs are synthetic analogues or mimetics of human GLP-1, which is an incretin glucoregulatory hormone released from the gut in response to food ingestion [5]. GLP-1RAs have multiple pleiotropic actions, as they bind to GLP-1 receptors expressed in many human tissues including the pancreas, kidneys, lungs, heart, brain, and gastrointestinal tract [5, 6]. GLP-1RAs reduce glycemia in patients with T2DM by increasing glucose-induced insulin secretion and inhibiting glucagon secretion via the stimulation of pancreatic GLP‐1 receptors in beta and alpha cells and by increasing insulin sensitivity [5]. GLP-1 and its analogues can also amplify insulin signaling in brain cells, leading to increased insulin sensitivity in neurons [7, 8].

Within the cardiovascular system, GLP-1 receptors are expressed on endothelial cells, monocytes, macrophages, and vascular smooth muscle cells (VSMCs) [9]. GLP-1 receptors are also widely expressed in the central nervous system, including the brainstem, cerebellum, hippocampus, cortex, hypothalamus, and amygdala [7, 10, 11]. There, the cellular expression of GLP-1 receptors is predominantly confined to neurons and dendrites [11].

GLP-1RAs are overall well-tolerated, with their most common adverse effects being nausea, vomiting, and diarrhea [7]. It has been recently shown that there are cholecystokinin-expressing neurons in the caudal brainstem, which are activated postprandially and are responsive to GLP-1RAs, explaining in part the body weight-lowering effects of GLP-1RAs but also their ability to induce nausea [12].

Based on similarities in their amino acid sequence, GLP-1RAs are peptide derivatives of either exendin-4 (exenatide, lixisenatide, and efpeglenatide) or human GLP-1 (albiglutide, dulaglutide, liraglutide, and semaglutide). Moreover, based on their pharmacokinetic/pharmacodynamic profile, GLP-1RAs can be classified into short-acting (exenatide and lixisenatide) and long-acting (albiglutide, dulaglutide, exenatide extended-release, liraglutide, semaglutide, and efpeglenatide) [5, 6].

The main pharmacokinetic difference between short-acting (half-life of 2–5 h) and long-acting (half-life > 12 h) GLP-1RAs is that short-acting GLP-1RAs are subject to wide fluctuations in the plasma concentration of the active compound, while long-acting GLP-1RAs exert a more constant effect on the GLP-1 receptor [13]. Furthermore, short-acting GLP-1RAs predominantly affect postprandial glucose levels, mainly by reducing gastric emptying speed, while long-acting GLP-1RAs more strongly affect fasting glucose levels through a combination of increased fasting insulin and reduced hepatic gluconeogenesis [14].

There are currently no head-to-head clinical trials comparing different GLP-1RAs. Nevertheless, in a network meta-analysis comparing short-acting and long-acting GLP-1RAs when used in conjunction with basal insulin, long-acting GLP-1RAs resulted in significantly greater reductions in glycated hemoglobin (HbA1c), fasting plasma glucose, and body weight compared to short-acting GLP-1RAs, with better gastrointestinal tolerability [15]. Regarding their protective effect against stroke, GLP-1RAs showed some heterogeneity in CVOTs, with the highest reduction in the risk of ischemic stroke reported for the long-acting GLP1-RA semaglutide at 28% [16] and no reduction for the short-acting lixisenatide [1].

All GLP-1RAs have been found to cross the blood–brain barrier (BBB) and stimulate GLP-1 receptors in the brain, thus offering neuroprotection [17]. However, exenatide is considered the most efficient in crossing the BBB based on its rate of brain influx, its percentage reaching the brain that accumulates in the brain parenchyma, and the percentage of the systemic dose taken up per gram of brain tissue [18].

## Extracerebral effects of glucagon-like peptide-1 receptor agonists

### Glycemic impact of GLP-1RAs

Hyperglycemia has a causal impact on increased risk of ischemic stroke [19]. In a meta-regression analysis of 18 CVOTs, including 8 with GLP-1RAs, a significant association between HbA1c reduction and major adverse cardiovascular events (including cardiovascular mortality, non-fatal myocardial infarction [MI], heart failure, and non-fatal stroke) was shown; this finding was almost driven by the association between HbA1c reduction and non-fatal stroke. For every 1% reduction in HbA1c (e.g., from 8 to 7%), the risk of non-fatal stroke decreased by 41% [20]. Similarly, in the REWIND (Researching cardiovascular Events with a Weekly INcretin in Diabetes) CVOT, an exploratory mediation analysis suggested that HbA1c reduction accounted for approximately 50% of the beneficial effects of dulaglutide on stroke [21]. Despite these findings, this association between HbA1c reduction and stroke risk reduction does not necessarily indicate that glycemic control is the sole actor in GLP-1RA-mediated stroke protection. It is possible that HbA1c reduction with GLP-1RAs could reflect other biological changes such as reduced insulin resistance, reduced visceral adiposity, and reduced inflammation, which could all be directly involved in GLP-1RA-mediated stroke protection.

### Blood pressure-lowering effects of GLP-1RAs

Hypertension is a leading risk factor for stroke. Accordingly, in patients with high blood pressure including those with T2DM, protection from stroke increases with the magnitude of blood pressure reduction [22]. An exploratory mediation analysis of stroke outcomes in the REWIND CVOT, in which dulaglutide reduced systolic blood pressure (SBP) by a mean of 1.7 mmHg compared to placebo, found that the blood pressure reduction caused by dulaglutide accounts for around 14% of its effect on stroke risk [21].

Although the mechanisms linking GLP-1 receptor activation to blood pressure control require further investigation in humans, GLP-1RAs have been shown to lower blood pressure by augmenting natriuresis and diuresis, which are mainly mediated by their inhibition of the Na^+^/H^+^ exchanger isoform 3, located at the brush border of the renal proximal tubule [23]. The natriuretic effect of GLP-1RAs may also be partially attributed to a reduction of the activity of the renin–angiotensin–aldosterone system, as in clinical studies, GLP-1 and GLP-1RAs have been found to lower circulating angiotensin II levels by approximately 20% [24, 25]. Additionally, in patients with T2DM, a 12-month GLP-1RA treatment was associated with increased endothelial glycocalyx thickness and reduced arterial stiffness, which could contribute to blood pressure reduction and stroke prevention [26]. Using a C57BL/6 mouse model, Kim and colleagues have further identified a gut-heart GLP-1 receptor-dependent and atrial natriuretic peptide-dependent axis that regulates blood pressure [27]. It remains however to be proven to which extent these described mechanisms are related to the modest blood pressure decrease observed in humans and whether they contribute to stroke prevention.

### Direct effects of GLP-1RAs on the heart

Diabetes mellitus is an established risk factor for atrial fibrillation (AF) [28]. Since dysrhythmia may cause contractile dysfunction and atrial blood stasis, patients with AF in turn face an elevated risk of stroke [29]. GLP-1RAs have been found to significantly reduce AF risk in patients with diabetes compared with placebo [30] and other glucose-lowering agents including metformin, sulfonylureas, and insulin [31]. The AF risk reduction by GLP-1RAs may be attributed to their protective effects on atrial electrical remodeling [28]. Indeed, in a canine AF model, liraglutide was found to suppress atrial electrophysiological changes such as AF inducibility and conduction velocity decrease [32]. Consistently, in a rat model of MI‑induced heart failure, exendin-4 decreased susceptibility to atrial arrhythmogenesis, improved conduction properties, and attenuated atrial fibrosis [33]. GLP-1RAs might also protect diabetic hearts from AF development through modulating calcium homeostasis. This was highlighted in an experimental study performed on GLP-1-treated HL-1 cardiomyocytes, in which GLP‑1 increased calcium transients and sarcoplasmic reticular calcium contents by regulating the expression of calcium handling proteins, leading to a reduced calcium leak and thus preventing AF development [34].

### Anti-hyperlipidemic effects of GLP-1RAs

In individuals with T2DM, lipid abnormalities are vital contributors to the risk of ASCVD, including the risk of ischemic stroke [35]. GLP-1RAs have shown modest beneficial effects on fasting plasma lipid levels in patients with T2DM [36, 37]. However, GLP-1RAs markedly reduced postprandial increases in triglycerides, apolipoprotein (Apo)B48 (major structural protein of chylomicrons), and ApoC-III (key regulator of triglyceride-rich lipoprotein metabolism), independently of gastric emptying [38–40]. Liraglutide has also been shown to significantly modify lipoprotein metabolism, with a reduction of chylomicron production [40, 41] and an increase in chylomicron and low-density lipoprotein (LDL) catabolism [40, 42]. In vitro studies further showed that liraglutide directly reduced the expression of several genes involved in chylomicron production and of the proprotein convertase subtilisin/kexin type 9 (*PCSK9*) gene [40, 42]. In a prospective, real-world, 4-month study performed in 62 patients with T2DM, liraglutide also reduced the number of small dense LDL (sdLDL) particles, known to be highly atherogenic [43]. sdLDL particles are more susceptible to oxidation than larger, more buoyant LDL particles. Hence, by reducing sdLDL, there is less substrate available to be oxidized, and thus liraglutide may help slow down or prevent the atherosclerotic processes at an early stage and prevent ischemic stroke [43].

### Anti-atherosclerotic effects of GLP-1RAs

Most ischemic strokes are caused by atherosclerosis. Vinué et al. showed in atherosclerosis-prone apolipoprotein E-deficient (ApoE^−^/^−^) and insulin-resistant mice that lixisenatide can diminish the atherosclerosis burden by reducing the size of atheroma plaques, increasing plaque stability, and reprogramming macrophages to the anti-inflammatory M2 phenotype by enhanced activation of signal transducer and activator of transcription (STAT)3, which is a determinant for M2 macrophage differentiation. On the other hand, STAT1 activation, which is essential for the M1 macrophage phenotype, was diminished [44]. Consistently, in another study of ApoE^−^/^−^ mice, anti-atherosclerotic effects of a liraglutide infusion included blocking uptake of oxidized LDL particles (potentially caused by a downregulation of the scavenger receptor CD36) and suppressing foam cell formation by reducing monocyte/macrophage infiltration in the aortic wall [45].

Besides the effects of GLP-1RAs on macrophage polarization into the M2 phenotype and on foam cell formation, GLP-1RAs may act directly on endothelial cells and limit endothelial dysfunction, which is primarily characterized by a reduction in nitric oxide (NO) bioavailability [46, 47]. In vitro/ex vivo studies conducted on endothelial cells showed that GLP-1RAs such as exenatide and liraglutide stimulate endothelial NO production and endothelial nitric oxide synthase (eNOS) activation, eliciting vasodilatation through activation of the GLP-1 receptor-dependent AMP-activated protein kinase (AMPK)/protein kinase B (Akt)/eNOS signaling pathway [48–50]. Moreover, GLP-1RAs also have anti-adhesive and anti-inflammatory properties in the vascular endothelium [47]. Under high glucose conditions, endothelial cells can acquire the characteristics of fibroblasts, via endothelial-mesenchymal transition (EndMT), which can contribute to neointimal hyperplasia [47]. In streptozotocin-induced diabetic mice, liraglutide treatment for 28 days was found to inhibit high glucose and interleukin (IL)-1β-induced EndMT in endothelial cells and consequently attenuate neointima formation via activation of the AMPK pathway, which was evidenced by a reduction of the expression of mesenchymal markers such as smooth muscle 22α (SM22α), vimentin, and Snail [51].

In addition to their endothelial protective properties, GLP-1RAs have been shown to delay atherogenesis by improving VSMC dysfunction. In an in vitro/ex vivo study, treatment of ApoE^−^/^−^ mice with liraglutide for 4 weeks dose-dependently inhibited angiotensin II-induced VSMC proliferation by activating AMPK signaling and inducing cell cycle arrest, thus delaying the progression of atherosclerosis, independently of its glucose-lowering effect [52]. VSMC senescence is another important aspect of VSMC dysfunction, being a feature of both atherosclerosis and plaque vulnerability [47]. In this regard, in an in vitro study by Zhou et al., exendin-4 suppressed angiotensin II-induced premature senescence of VSMCs by inhibiting superoxide production through activation of nuclear factor-erythroid-2–related factor 2 (Nrf2) [53]. VSMC phenotype transition is also involved in diabetes-associated ASCVDs such as stroke [47]. In rat coronary artery smooth muscle cells, treatment with liraglutide inhibited VSMC phenotypic transition induced by advanced glycation end products (AGEs), through blocking the nuclear factor-κB (NF-κB) signaling pathway and activating the protein kinase A (PKA) signaling pathway. Liraglutide also increased the expression of VSMC contractile markers such as α-smooth muscle actin (α-SMA), smooth muscle myosin heavy chain 11 (MYH11), and myocardin [54].

Studies in humans have mostly confirmed the anti-atherosclerotic actions of GLP-1RAs. For instance, in an 18-month prospective, real-world study conducted in 121 patients with T2DM and metabolic syndrome, liraglutide treatment was associated with a significant reduction in carotid intima-media thickness, a surrogate marker of subclinical atherosclerosis, from a mean of 0.97 mm at baseline to 0.78 mm at 18 months [55]. There are also some clinical studies indicating that GLP-1RA therapy might attenuate the inflammatory cascade leading to the development of atherosclerosis [56]. For example, in a small trial of 10 patients with T2DM, liraglutide therapy for 8 weeks reduced the expression of the inflammatory macrophage activation molecule, soluble CD163, a biomarker for accelerated atherosclerosis, by 22% (p < 0.001 versus baseline), independently of its glucose-lowering effect [57]. Balestrieri et al. also evaluated the effect of incretin-based therapy (either GLP-1RAs or DPP-4i) on atherosclerotic plaques obtained from 52 patients with T2DM (of whom 24 were treated with GLP-1RAs or DPP-4i for a mean duration of 26 months) and 30 non-diabetic patients undergoing carotid endarterectomy [58]. Compared with non-diabetic plaques, diabetic plaques had more inflammation and oxidative stress, along with a lower sirtuin (SIRT)6 expression and less interstitial collagen content. More importantly, compared with non-GLP-1RA/DPP-4i-treated plaques, GLP-1RA/DPP-4i-treated plaques presented greater SIRT6 expression and collagen content, with less inflammation and oxidative stress, indicating a more stable plaque phenotype and suggesting that GLP-1RAs can reduce plaque vulnerability [58].

## Cerebral effects of glucagon-like peptide-1 receptor agonists

Independently of the aforementioned peripheral actions, GLP-1RAs also have direct cerebral effects that may contribute to reducing the risk of stroke in patients with T2DM. A recent proteomic analysis revealed that GLP-1RAs exert various effects on the cerebral expression of proteins in mice subjected to middle cerebral artery occlusion (MCAO), with 17 upregulated and 10 downregulated proteins [59]. Specifically, GLP-1RA administration downregulated the protein expression of haptoglobin, upregulated levels of PRKC apoptosis WT1 regulator (PAWR) and of synapsis-related proteins including synapsin-1, phosphodiesterase-2A (PDE2A), dihydropyrimidinase-like 2 (DPYSL2), neurofibromin-1, and microtubule-associated protein 1B (MAP1B), and increased neuronal and synaptic densities [59]. Several animal studies have further shown that administration of GLP-1RAs, before or shortly after experimentally-induced stroke, increased angiogenesis, neurogenesis and cerebral blood flow (CBF), reduced neuroinflammation, oxidative stress, excitotoxicity, BBB leakage and apoptosis, and induced dose-dependent decreases in infarct volume [60–62]. In addition, administration of GLP-1RAs in experimental stroke was found to activate several intracellular signaling pathways involved in neuroprotection, including cyclic adenosine monophosphate (cAMP)/PKA/cAMP-response element binding protein (CREB); phosphatidylinositol-3 kinase (PI3K)/Akt; mitogen-activated protein kinase (MAPK)/extracellular signal-regulated kinase (ERK); Wnt/β-catenin; and Nrf2/heme oxygenase-1 (HO-1) [60, 62–64].

### Effects of GLP-1RAs on angiogenesis, CBF, and infarct volume

In mice with focal cerebral cortical ischemia induced by MCAO, intraperitoneal administration of once-daily liraglutide (for 14 days), 24 h following stroke induction, promoted angiogenesis through significantly increasing the expression of vascular endothelial growth factor (VEGF) in cerebral ischemic areas as compared to normal saline treatment [65]. Similarly, Sato et al. [66] demonstrated upregulation of VEGF in the cerebral cortex of liraglutide-treated rats subjected to MCAO; however, this was not seen within the striatum [66]. In another rat model of ischemic stroke, liraglutide treatment promoted neurovascular remodeling in cerebral ischemic areas, through induction of angiogenesis and an increase in neural cell activity [67]. GLP-1RA-induced angiogenesis appears to be mediated through the PI3K/Akt, PKA, and Src pathways [68].

GLP-1RAs may also offer neuroprotection by improving/maintaining CBF in the regions surrounding the affected brain area [69]. In a recent in vivo/ex vivo study using a rat model of ischemic stroke induced by MCAO, systemic administration of exendin-4 was found to be neuroprotective via its vasodilatory action on cortical arterioles and improved CBF [69]. Data accumulated from animal experiments conducted in acute ischemic stroke models with and without diabetes have also demonstrated a dose-dependent reduction of infarct volume in the brain by GLP-1RAs, when administered before or during the acute phase of ischemia at reperfusion or with some delay after the onset of reperfusion [60–62, 70].

### Anti-neuroinflammatory effects of GLP-1RAs

GLP-1RAs, administered following stroke induction in animal studies, have been associated with an anti-inflammatory effect [62]. In rodent models, lixisenatide, liraglutide, and exenatide have been reported to significantly reduce brain levels of the pro-inflammatory cytokine, tumor necrosis factor α (TNF-α) [71–73]. Reduction of other inflammatory biomarkers has also been reported following GLP-1RA treatment in rodent models of cerebral ischemia, namely myeloperoxidase, IL-1β, IL-6, IL-18, and cyclooxygenase-2 (COX-2), the latter through increasing expression of islet-brain-1 (IB1) [72–74]. Exendin-4 has been further found to decrease the expression of hypoxia-inducible factor-1α (HIF-1α) in the gerbil hippocampus after global brain ischemia [75], which stimulates inflammatory cytokines’ expression [60]. Moreover, in both young and aged diabetic mice subjected to MCAO, exendin-4 treatment was shown to significantly polarize microglia/macrophages towards an anti-inflammatory M2 phenotype in the injured hemisphere compared to the non-injured hemisphere [76]. Hence, GLP-1RAs can attenuate neuroinflammation through microglial M2 polarization [76].

Another pro-inflammatory target of GLP-1RAs is matrix metalloproteinase-9 (MMP-9), an enzyme that increases BBB permeability and promotes BBB breakdown through proteolytic activity [77]. In mice with transient hyperglycemic and acute focal ischemia induced by MCAO, exendin-4, but not insulin, reduced the expression of MMP-9 and consequently BBB permeability. Exendin-4 also reduced vascular immunoglobulin G extravasation, indicating reduced endothelial leakage in the late inflammatory response to ischemia [71]. Consistently, in another in vivo/in vitro study using a rat transient MCAO model, exendin-4 reduced BBB tight-junction protein degradation by inhibiting MMP-9 activation and reactive oxygen species (ROS) production via activation of the Wnt/β-catenin signaling pathway [64]. Exendin-4 also appears to preserve the integrity of the BBB after cerebral ischemia by inactivating glycogen synthase kinase-3β (GSK-3β), a serine/threonine protein kinase, through the PI3K/Akt pathway [78].

These animal data are in line with findings from human studies [57, 79–81]. For instance, in a placebo-controlled study of 24 obese patients with T2DM, exenatide treatment for 12 weeks exerted an anti-inflammatory effect independent of body weight reduction, as illustrated by reduced circulating levels of various pro-inflammatory mediators such as TNF-α, IL-1β, IL-6, serum amyloid A, and MMP-9 [79]. In another study of patients with T2DM and obesity, 8-week liraglutide therapy was associated with a decrease in levels of the pro-inflammatory cytokines TNF-α, IL-1β, and IL-6, together with an increase in levels of the anti-inflammatory adipokine, adiponectin, in peripheral blood mononuclear cells. These changes were independent of reductions in body weight and in glycemic control markers, such as fructosamine and HbA1c [57]. Consistently, in a more recent meta-analysis of 40 randomized controlled trials performed in patients with T2DM, compared with standard diabetes therapies (i.e., metformin, sulfonylureas, insulin, DPP-4i, thiazolidinediones) and placebo, GLP-1RA therapy was associated with significant reductions in inflammatory markers such as serum C-reactive protein (CRP) and TNF-α, and a significant increase in adiponectin [80].

Given that brain inflammation is an immune response mediated by microglia and astrocytes, it has been postulated that the anti-neuroinflammatory effect of GLP-1RAs could be in part due to their inhibitory action on astrocytes and microglia via the activation of cAMP/PKA signaling [82]. Activation of the GLP-1 receptor-mediated cAMP/PKA pathway is also involved in the protection of astrocytes from AGE-induced inflammatory response and from inflammatory cytokine secretion [83, 84].

### Effects of GLP-1RAs on oxidative stress and mitochondrial function

In patients with diabetes, chronic hyperglycemia can lead to excessive production of ROS and consequent imbalance in redox status in brain tissues, which may play an important role in the pathogenesis of ischemic stroke [85]. Several animal studies conducted in diabetic and non-diabetic rodent models of focal cerebral ischemia reported an improvement in redox status parameters following administration of exenatide, liraglutide, or lixisenatide, with reduced levels of cerebral malondialdehyde (MDA) and increased cerebral concentrations of glutathione and superoxide dismutase (SOD) [73, 85–87]. GLP-1RAs also reduced several other markers of oxidative stress in rodent stroke models, such as blood and brain ROS counts, blood levels of derivatives of reactive oxygen metabolites (d-ROMs), and brain levels of dihydroethidium, a marker of ROS production [66, 87]. Similarly, in the clinical setting, GLP-1RA treatment was associated with reduced oxidative stress markers such as 8-iso-prostaglandine-F2α (8-iso-PGF2α), d-ROMs, superoxide, MDA, and lipid peroxides [80, 88, 89].

The mechanisms by which GLP-1RAs reduce oxidative stress in the brain have not been well-elucidated. However, in an in vivo study performed in diabetic rats with brain ischemia, the anti-oxidant effects of liraglutide treatment (i.e., increased SOD and reduced myeloperoxidase levels) were mediated by activation of the mitochondrial ATP-sensitive potassium channel composed of sulfonylurea receptor 1 (SUR1) and the K^+^-selective inward rectifier Kir6.2 [90]. Consistently, in an in vivo/in vitro study using a focal cerebral cortical ischemic mouse model and cultures of cortical neurons under oxidative stress, liraglutide promoted brain repair after cerebral ischemic injury and reduced oxidative stress, through SIRT1‐mediated mitochondrial improvement [91].

### Anti-apoptotic effects of GLP-1RAs

In both diabetic and non-diabetic rodent models of cerebral ischemia, exenatide, lixisenatide, semaglutide, and liraglutide increased the expression of the anti-apoptotic factor B-cell lymphoma 2 (Bcl-2) and decreased the expression of the pro-apoptotic factor Bcl-2-associated X protein (Bax), consequently leading to a reduced Bax/Bcl-2 ratio and reduced apoptosis, possibly through ROS reduction and activation of the PI3K/Akt and MAPK pathways [73, 86, 87, 92, 93]. Expression of cerebral caspase-3, involved in apoptosis, was also consistently reduced by exenatide, liraglutide, lixisenatide, and semaglutide in stroke animal models [73, 85, 87, 92, 93]. Exenatide and liraglutide additionally lowered the expression of other apoptosis-related proteins, including poly (ADP-ribose) polymerase (PARP), caspase-8, caspase-9, and Bcl-2-associated death promoter (Bad) [87, 92]. More recently, an in vivo/in vitro study using a mouse model of focal cerebral cortical ischemia revealed that the neuroprotective effects of liraglutide (i.e., reduced brain infarct volume, improved neurological recovery, and anti-neuroinflammatory effects) may be achieved through the inhibition of pyroptosis, which is special form of apoptosis associated with inflammation and mediated by inflammasome and caspase-1 activation [94]. More specifically, the primary target of the anti-pyroptotic effect of liraglutide was identified as the NOD-like receptor protein 3 (NLRP3) inflammasome [94].

### Effects of GLP-1RAs on excitotoxicity

Excitotoxicity is a primary mechanism of neuronal injury following stroke, which can be attributed to the excessive activation of glutamate receptors. This in turn can lead to intracellular calcium overload that is particularly neurotoxic, ultimately resulting in the degradation of proteins, membranes, and nucleic acids [95]. When examining the neurotrophic properties of GLP-1 and exenatide on cultured hippocampal neurons and in a rodent model of neurodegeneration, treatment with GLP-1 and exendin-4 was found to prevent and reverse excitotoxic neuronal damage, as measured by increased cholinergic marker activity [96]. The production of brain-derived neurotrophic factor (BDNF) in a CREB-dependent manner, which can prevent excitotoxic neuronal death by reducing the neurotoxic release of glutamate [60], has also been found to be increased following exenatide treatment in adult wild-type mice [97]. On that same note, it was recently demonstrated that a generalized reversal of functionally relevant transcriptomic changes at the genome-wide level in multiple glial and vascular cell types of the brain is pharmacologically achievable with GLP-1RA treatment [98]. Hence, GLP-1RAs may offer the possibility for rescue of damaged neurons in the brain associated with both neurodegeneration and ischemic stroke.

### Effects of GLP-1RAs on neurogenesis and neuroplasticity

Neurogenesis, consisting in the formation of new neurons from neural stem and progenitor cells located in various brain regions, such as the subgranular zone of the dentate gyrus in the hippocampus and the subventricular zone of lateral ventricles, has the potential to reduce neuronal damage and restore neurological function after a stroke [93]. In a rat model of MCAO-induced stroke, neurogenesis was increased by semaglutide treatment, as evidenced by significantly increased levels of neurogenesis-specific biomarkers including nestin (intermediate filament protein that is a known marker of neuronal progenitor cells), doublecortin (microtubule-associated protein considered to be a reliable marker of neurogenesis), stromal cell-derived factor-1α (SDF-1α) (chemokine promoting endogenous regeneration in the ischemic brain), and its receptor CXCR4. The number of doublecortin-positive cells in the dentate gyrus was also increased with semaglutide treatment [93]. It has been postulated that the restoration of insulin signaling sensitivity by GLP-1RA treatment and improved growth factor signaling, as shown by increased levels of activated ERK1 and insulin receptor substrate-1 (IRS1) following semaglutide treatment, is responsible for the normalization of stem cell proliferation and neurogenesis in the brain [93].

GLP-1RAs have been further found to be effective in improving neuroplasticity, which may also promote functional recovery post-stroke [8, 99, 100]. This was highlighted in a recent study performed in a mouse model of obesity-induced T2DM, in which an 8-week exendin-4 treatment, starting 3 days post-stroke, improved neurological recovery by reversing T2DM-induced atrophy of parvalbumin-positive GABAergic interneurons, which play a key role in neuroplasticity [8]. The improved neuroplasticity resulting from GLP-1RA therapy appears to be mediated by GLP-1 receptors in the hippocampus. More specifically, an in vivo study, based on chronic 40-day peripheral administration of lixisenatide to high-fat fed mice with established obesity, insulin resistance and impaired cognition, found that lixisenatide significantly upregulated expression of the neurotrophic tyrosine kinase receptor type 2 (*NTRK2*) gene and of the mammalian target of rapamycin (*mTOR*) gene in the hippocampus, which are involved in regulating synaptic plasticity [100].

## Conclusions

**Fig. 1:**
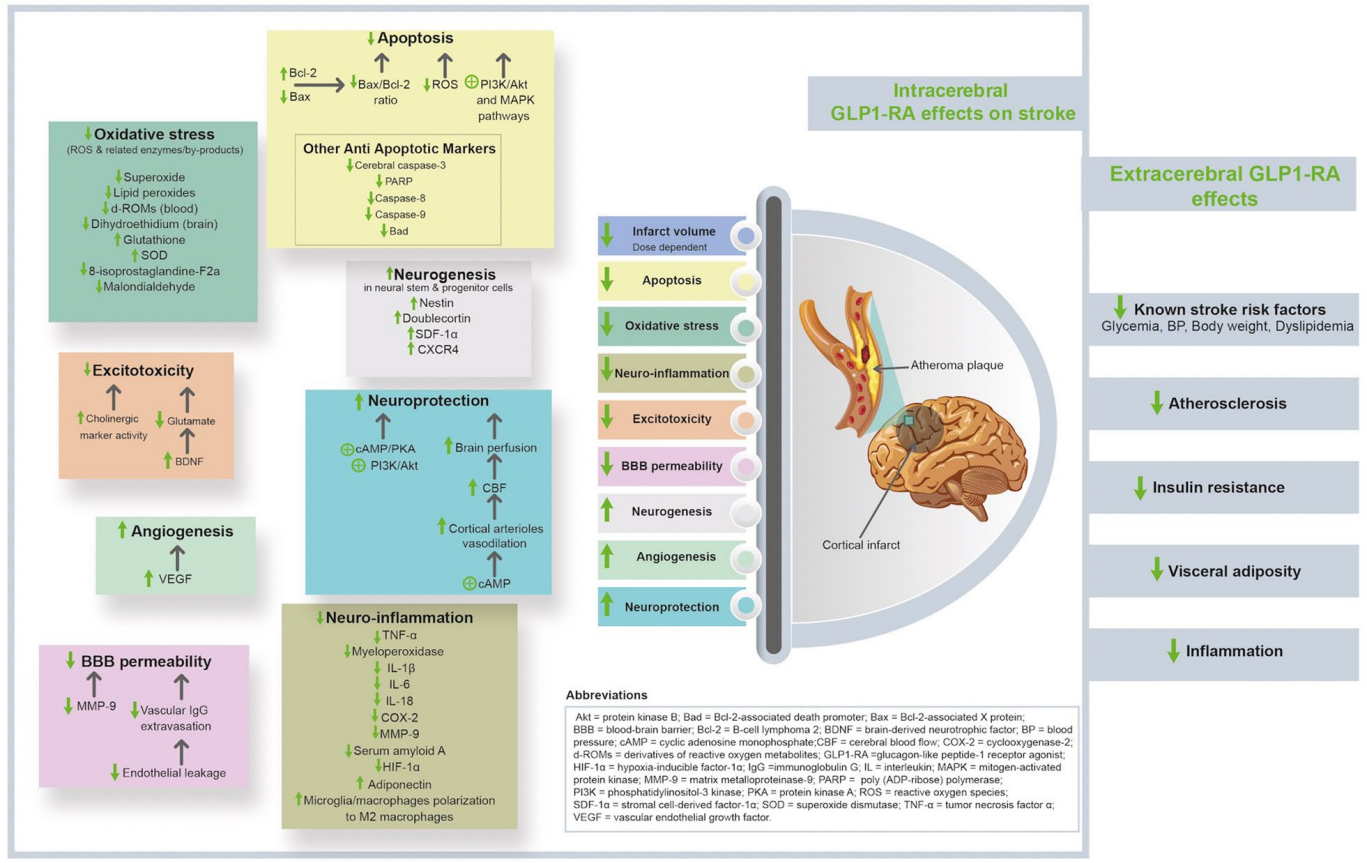
Potential mechanisms of stroke protection mediated by glucagon-like peptide-1 receptor agonists, with a focus on their intracerebral effects

A series of mechanisms can potentially explain how GLP-1RAs may contribute to reduce the risk of stroke and promote brain protection in patients with T2DM (Fig. 1). In animal stroke models, GLP-1RAs have been associated with various beneficial direct cerebral effects, such as reduced infarct volume, apoptosis, oxidative stress, mitochondrial dysfunction, neuroinflammation, excitotoxicity and BBB permeability, and increased neurogenesis, neuroplasticity, angiogenesis and CBF. GLP-1RAs have also demonstrated several direct anti-atherosclerotic effects such as reduced VSMC dysfunction, increased NO, reduced vascular inflammation, and improved endothelial function. GLP-1RAs further lower the risk of stroke indirectly by reducing traditional stroke risk factors such as HbA1c, SBP, body weight, and dyslipidemia. The available evidence remains however insufficient to confirm whether other effects of GLP-1RAs such as direct cardioprotective actions (i.e., effects on atrial electrical remodeling, increased left ventricular ejection fraction, increased myocardial salvage, effects on cardiac oxidative metabolism) and effects on the microvascular function may mechanistically contribute to GLP-1RA-mediated protection against stroke.

Although our review highlights several promising effects of GLP-1RAs in the brain, there are some limitations that should be acknowledged. Only very limited studies reported negative results on the anti-stroke effects of GLP-1 and GLP-1RAs, which, along publication bias, favor positive outcomes. Moreover, the non-clinical studies were mainly conducted in homogeneous rodent models, limiting their representation of human stroke, which is a highly heterogeneous, multifactorial disorder [101]. Infarct progression in the human brain is also presumed to be 2–3 times slower than in the rodent brain [70]. In addition, given that most non-clinical studies evaluating the anti-stroke effects of GLP-1RAs were performed in animal models of acute ischemic stroke, in which GLP-1 or its analogues were administered before, during, or after stroke induction, the mechanistic effects of GLP-1RAs in preventing stroke on the long-term need further investigation. To date, most clinical studies investigating the anti-stroke effects of GLP-1RAs estimated the risk of an ischemic stroke event, without focusing on mechanistic or functional outcomes. Hence, further research is still needed to understand the most important mechanisms involved in GLP-1RA-mediated stroke protection specifically in the human diabetic brain and to connect the described experimental studies to clinical stroke. There are currently two ongoing randomized, phase II studies (NCT02829502; NCT03287076) performed in patients with ischemic stroke to evaluate the impact of exenatide therapy on various mechanistic outcomes such as CBF velocity, endothelial function, inflammation, and post-stroke hyperglycemia. When available, the results of these two clinical studies may enhance our understanding of the mechanisms of stroke protection by GLP-1RAs in humans.

## Data Availability

Not applicable.

## Abbreviations

8-iso-PGF2α: 8-Iso-prostaglandine-F2α
α-SMA: α-Smooth muscle actin
AF: Atrial fibrillation
AGE: Advanced glycation end product
Akt: Protein kinase B
AMPK: AMP-activated protein kinase
Apo: Apolipoprotein
ApoE^−^/^−^: Apolipoprotein E-deficient
ASCVD: Atherosclerotic cardiovascular disease
Bad: Bcl-2-associated death promoter
Bax: Bcl-2-associated X protein
BBB: Blood–brain barrier
Bcl-2: B-cell lymphoma 2
BDNF: Brain-derived neurotrophic factor
cAMP: Cyclic adenosine monophosphate
CBF: Cerebral blood flow
COX-2: Cyclooxygenase-2
CREB: CAMP-response element binding protein
CRP: C-reactive protein
CVOT: Cardiovascular outcome trial
DPP-4: Dipeptidyl peptidase-4
DPYSL2: Dihydropyrimidinase-like 2
d-ROMs: Derivatives of reactive oxygen metabolites
EndMT: Endothelial-mesenchymal transition
eNOS: Endothelial nitric oxide synthase
ERK: Extracellular signal-regulated kinase
GLP-1RA: Glucagon-like peptide-1 receptor agonist
GSK-3β: Glycogen synthase kinase-3β
HbA1c: Glycated hemoglobin
HIF-1α: Hypoxia-inducible factor-1α
HO-1: Heme oxygenase-1
IB1: Islet-brain-1
IL: Interleukin
IRS1: Insulin receptor substrate-1
LDL: Low-density lipoprotein
MAP1B: Microtubule-associated protein 1B
MAPK: Mitogen-activated protein kinase
MCAO: Middle cerebral artery occlusion
MDA: Malondialdehyde
MI: Myocardial infarction
MMP-9: Matrix metalloproteinase-9
mTOR: Mammalian target of rapamycin
MYH11: Smooth muscle myosin heavy chain 11
NF-κB: Nuclear factor-κB
NLRP3: NOD-like receptor protein 3
NO: Nitric oxide
Nrf2: Nuclear factor-erythroid-2–related factor 2
NTRK2: Neurotrophic tyrosine kinase receptor type 2
PARP: Poly (ADP-ribose) polymerase
PAWR: PRKC apoptosis WT1 regulator
PCSK9: Proprotein convertase subtilisin/kexin type 9
PDE2A: Phosphodiesterase-2A
PI3K: Phosphatidylinositol-3 kinase
PKA: Protein kinase A
REWIND: Researching cardiovascular Events with a Weekly INcretin in Diabetes
ROS: Reactive oxygen species
SBP: Systolic blood pressure
SDF-1α: Stromal cell-derived factor-1α
sdLDL: Small dense LDL
SIRT: Sirtuin
SM22α: Smooth muscle 22α
SOD: Superoxide dismutase
STAT: Signal transducer and activator of transcription
SUR1: Sulfonylurea receptor 1
T2DM: Type 2 diabetes mellitus
TNF-α: Tumor necrosis factor α
VEGF: Vascular endothelial growth factor
VSMC: Vascular smooth muscle cell
